# Directed Differentiation of Patient-Specific Induced Pluripotent Stem Cells Identifies the Transcriptional Repression and Epigenetic Modification of NKX2-5, HAND1, and NOTCH1 in Hypoplastic Left Heart Syndrome

**DOI:** 10.1371/journal.pone.0102796

**Published:** 2014-07-22

**Authors:** Junko Kobayashi, Masashi Yoshida, Suguru Tarui, Masataka Hirata, Yusuke Nagai, Shingo Kasahara, Keiji Naruse, Hiroshi Ito, Shunji Sano, Hidemasa Oh

**Affiliations:** 1 Department of Cardiovascular Surgery, Okayama University Graduate School of Medicine, Dentistry, and Pharmaceutical Sciences, Okayama, Japan; 2 Department of Cardiovascular Medicine, Okayama University Graduate School of Medicine, Dentistry, and Pharmaceutical Sciences, Okayama, Japan; 3 Department of Cardiovascular Physiology, Okayama University Graduate School of Medicine, Dentistry, and Pharmaceutical Sciences, Okayama, Japan; 4 Department of Regenerative Medicine, Center for Innovative Clinical Medicine, Okayama University Hospital, Okayama, Japan; Tokai University, Japan

## Abstract

The genetic basis of hypoplastic left heart syndrome (HLHS) remains unknown, and the lack of animal models to reconstitute the cardiac maldevelopment has hampered the study of this disease. This study investigated the altered control of transcriptional and epigenetic programs that may affect the development of HLHS by using disease-specific induced pluripotent stem (iPS) cells. Cardiac progenitor cells (CPCs) were isolated from patients with congenital heart diseases to generate patient-specific iPS cells. Comparative gene expression analysis of HLHS- and biventricle (BV) heart-derived iPS cells was performed to dissect the complex genetic circuits that may promote the disease phenotype. Both HLHS- and BV heart-derived CPCs were reprogrammed to generate disease-specific iPS cells, which showed characteristic human embryonic stem cell signatures, expressed pluripotency markers, and could give rise to cardiomyocytes. However, HLHS-iPS cells exhibited lower cardiomyogenic differentiation potential than BV-iPS cells. Quantitative gene expression analysis demonstrated that HLHS-derived iPS cells showed transcriptional repression of NKX2-5, reduced levels of TBX2 and NOTCH/HEY signaling, and inhibited HAND1/2 transcripts compared with control cells. Although both HLHS-derived CPCs and iPS cells showed reduced *SRE* and *TNNT2* transcriptional activation compared with BV-derived cells, co-transfection of NKX2-5, HAND1, and NOTCH1 into HLHS-derived cells resulted in synergistic restoration of these promoters activation. Notably, gain- and loss-of-function studies revealed that NKX2-5 had a predominant impact on *NPPA* transcriptional activation. Moreover, differentiated HLHS-derived iPS cells showed reduced H3K4 dimethylation as well as histone H3 acetylation but increased H3K27 trimethylation to inhibit transcriptional activation on the *NKX2-5* promoter. These findings suggest that patient-specific iPS cells may provide molecular insights into complex transcriptional and epigenetic mechanisms, at least in part, through combinatorial expression of NKX2-5, HAND1, and NOTCH1 that coordinately contribute to cardiac malformations in HLHS.

## Introduction

Single ventricle (SV) physiology including hypoplastic left heart syndrome (HLHS) is characterized by obstruction of the left ventricle (LV) in association with absence or underdevelopment of the left ventricular chamber morphogenesis that is not capable of supporting systemic cardiac output. Infants with SV physiology theoretically undergo two major medical treatments: cardiac transplantation and three-stage palliative reconstruction [1], [2]. However, their long-term survival has been shown to be remarkably lower than that of patients with all other congenital heart diseases [3].

Embryonic development in the heart is controlled by a core set of essential cardiac transcription factors that regulate progenitor cell migration and expansion, morphogenesis of cardiac chambers, and maturation of structural proteins. Genetically, HLHS has been associated with chromosome anomalies, but no single genetic basis has been found to be specifically linked to this syndrome [4]. Although the majority of cases are sporadic, rare patients who inherited multiple gene variants suggest that HLHS may be pathophysiologically heterogeneous and caused by cumulative effects that are poorly understood [5].

Obviously, most of the disease-causing genes for HLHS remain to be identified; however, the rarity of kindred with familial recurrences of HLHS with viable penetrance and phenotype has made linkage studies difficult. Patient-derived cardiac progenitor cells (CPCs) may allow researchers to investigate early cardiac development programs that have already acquired the disease phenotypes. A simultaneous tractable system, reported here, may facilitate the identification of crucial determinants in HLHS by modeling the disease with reprogramming technology that enables comprehensive analysis of patient-specific genetic disorders prior to disease onset.

iPS cells, which closely resemble embryonic stem (ES) cells, can be derived from human somatic tissues from a variety of diseases to recapitulate complex physiological phenotypes. Patient-specific iPS cells may provide an additional tool for studying human heart disease [6]. Here, to identify the key elements responsible for hypoplasia of left heart development, we generated disease-specific iPS cells in patients with congenital heart malformation, identified unique genes differentially expressed in HLHS, and explored the responsible regulatory network involved in myocardial patterning and morphogenesis during cardiac development with respect to LV hypoplasia in humans.

## Materials and Methods

### Ethics Statement

Written informed consent was obtained from the parents of the patients before the collection of tissue samples. The study protocols using human tissue samples and animals were approved by the Research Ethics Committee of Okayama University. As control RNA of human embryonic stem cells, khESC-1-derived RNA was isolated and provided by Keio University as previously reported [7].

### Tissue Sample and Cell Culture

Myocardial tissues were obtained from right atria during open-heart surgery shortly after cardiopulmonary bypass. Cardiac specimens were digested using 0.4% type II collagenase and 0.01% DNAse and minced to produce a single-cell suspension. The cells were seeded at 20 cells/µl in ultra-low culture dishes to generate cardiospheres with growth medium containing DMEM/F12 (Invitrogen), 10% fetal bovine serum, 20 ng/ml recombinant human epidermal growth factor (EGF; Wako), 40 ng/ml recombinant human basic fibroblast growth factor (bFGF; Wako), 50 U/ml penicillin, and 50 mg/ml streptomycin as previously described [8], [9]. Cardiospheres were mechanically harvested, digested, and cultured on poly-D-lysine-coated dishes to obtain CPCs.

### Generation and Differentiation of Patient-Specific iPS Cells

For iPS cell generation, CPCs were infected with concentrated retroviruses pseudotyped with vesicular stomatitis virus surface protein (VSV-G; Cell Biolabs, Inc.) encoding the human transcription factors OCT4, KLF4, SOX2, and MYC (Addgene) as previously described [10]. Transfected CPCs were maintained on mouse SNL feeders for 7 days post-infection and then cultured in DMEM/F12 containing 20% Knockout Serum Replacement, 2 mM L-glutamine, 0.1 mM nonessential amino acid (NEAA), 0.1 mM 2-mercaptoethanol (2-ME), 50 U/mL penicillin, 50 mg/ml streptomycin (all from Invitrogen), and 4 ng/mL recombinant human bFGF until iPS colonies appeared. Control human iPS cells (201B7; HPS0063) were purchased from RIKEN Bioresource Center.

Cardiac differentiation was induced on Matrigel-coated culture dishes in feeder-free conditions. The medium was replaced with serum-free medium (RPMI1640, Invitrogen) containing B27 (RPMI/B27, Invitrogen) and supplemented with 100 ng/ml human recombinant activin A for 24 h, followed by incubation with 10 ng/ml human recombinant bone morphogenetic protein (BMP) 4 for an additional 4 days (all from R&D Systems).

### Immunofluorescence

Cells were fixed in 4% paraformaldehyde for 30 min and stained in blocking buffer with primary antibodies against NANOG (R&D Systems), SSEA-3 (BD Biosciences), OCT4, SSEA-4, TRA-1-60, and TRA-1-81 (all from Millipore), followed by incubation with Alexa Fluor 488/546/594 secondary antibodies (Molecular Probes). To evaluate cardiomyocyte differentiation, fixed cells were permeabilized with 0.2% Triton X-100 for 30 min and incubated with primary antibodies directed against cardiac troponin-T (Abcam). Cells were counterstained with DAPI (Molecular Probes) and visualized using a confocal laser scanning microscope (FV-1000, Olympus, Tokyo). Alkaline phosphatase staining was performed using Alkaline Phosphatase Detection kit (Millipore) according to the manufacturer's instructions.

### Bisulfite Sequencing and Karyotype Analysis

Genomic DNA (1 µg) was extracted (QIAGEN) from patient-derived undifferentiated CPCs or iPS cells and processed for bisulfite conversion by using a Methylamp DNA modification kit (Epigentek). The promoter regions of OCT4 and NANOG were then amplified by PCR using primers as previously described (Table S2 in File S1) [11]. The PCR products were subcloned into pCRII-TOPO vector (Invitrogen) and analyzed using ABI 3700 DNA analyzer (Applied Biosystems). Chromosomal analysis was performed using the standard G-banding chromosome detection method (SRL).

### Teratoma Formation

Disease-specific iPS cells (0.6–1×10^6^ cells) were directly injected into the testes of NOD/SCID mice. At 10 to 12 weeks after transplantation, teratomas were dissected and processed for paraffin embedding and hematoxylin and eosin staining.

### Real-Time RT-PCR and Global Gene Expression Analysis

Total RNA was isolated using TRIzol reagent (Invtrogen) and cDNA was synthesized using Reverse Transcription Kit (QIAGEN). Quantitative RT-PCR was performed using FastStart Universal Probe Master (Roche) analyzed with the 7300 real-time PCR system (Applied Biosystems) and primers. All experiments were conducted more than five times with three different clonal derivatives. PCR was carried out using Ex Taq (Takara). All data were normalized using β2-microglobulin (B2M) and human heart tissue for comparisons. Primer sequences are shown in Table S2 in File S1. To assess the transcriptional profile, cyanine-labeled antisense RNA was amplified and hybridized with a Whole Human Genome Microarray (Agilent). Data were analyzed using GeneSpring GX10.0 software and gene expression levels were normalized using Robust Multi-array Average algorithm.

### ChIP Assay

The experiments of chromatin immunoprecipitation (ChIP) were performed using ChIP-IT Express Enzymatic Magnetic Chromatin Immunoprecipitation kit (Active Motif). Briefly, 1×10^6^ CPCs, undifferentiated iPS cells, and differentiated iPS cells were cross-linked with 1% formaldehyde solution for 5 min at room temperature. The cells were harvested and nuclei were extracted, lysed, and enzymatically sheared to obtain chromatin. Immunoprecipitation was performed on an end-to-end rotation overnight at 4°C using the following antibodies: IgG (Upstate, Millipore 12-370), H3K4me2 (Upstate, Millipore 07-030), H3K27me3 (Upstate, Millipore 07-449), and acH3 (Upstate, Millipore 06-599). Crosslinking between DNA and proteins was reversed. DNA was purified after proteinase K digestion using Chromatin IP DNA Purification Kit (Active Motif). All precipitated DNA samples were amplified and quantitated by real-time PCR with SYBR Premix Ex Taq II (TaKaRa) and NKX2-5 ChIP primers (Table S2 in File S1). Signals corresponding to each antibody were normalized by respective input.

### Promoter Assays

The TNNT2 (HPRM12846-PG04) and NPPA (natriuretic peptide A; HPRM23486-PG04) promoter activities were measured using a Dual Luminescence Assay Kit with a dual reporter construct containing Gaussia luciferase (GLuc) and secreted alkaline phosphatase (SEAP) side by side from a single sample (all from GeneCopoeia, Inc.). Each promoter is placed upstream of the GLuc reporter gene and contains a specific cardiac transcription factor as an insert. A secondary reporter gene SEAP was used to monitor the transfection efficiency for normalization. To detect serum response element (SRE) promoter activities, both SRE fragment (pGL4.33 [luc2P/SRE/Hygro], Promega) and pGL4.74 (hRluc/TK, Promega) were co-transfected into human cells by using X-tremeGene HP reagent (Roche). The relative expression of SRE (luc2P) construct was normalized using a control vector (hRluc) introduced into the same cells. Human cells were transfected with human NKX2-5 cDNA (SC122678), human NKX2-5 shRNA (short hairpin RNA; TR311165B), human HAND1 cDNA (SC122690), human HAND1 shRNA (TR316857C), human NOTCH1 cDNA (SC308883), or human NOTCH1 shRNA (TR302916D) along with single- or dual-reporter constructs (all from OriGene Technologies, Inc.). The luciferase activities were measured by the Glomax-Multi+Detection System (Promega) 48 hours after transfection.

### Statistics

Results are presented as the mean ± S.D. The significance of differences was evaluated by paired or unpaired Student's *t* test. A *p* value of less than 0.05 was considered significant.

## Results

### Generation of Disease-Specific iPS Cells Using Patient-Derived CPCs

**Figure 1:**
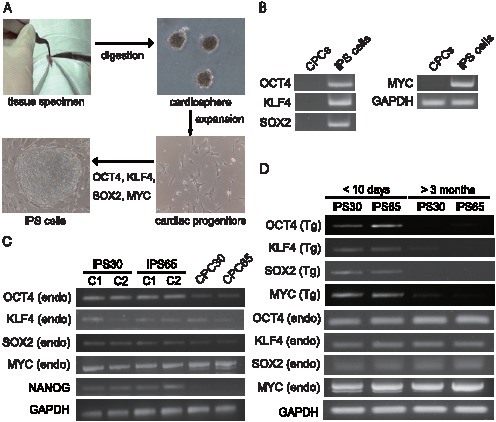
Reprogramming of disease-specific CPCs. (A) Schematic presentation of CPC isolation and iPS generation. (B) Retroviral transduction was verified by tagged-PCR and endogenous genes (C) are shown. (D) Retroviral silencing was confirmed during reprogramming after 3 months of infection. Trans- and endogenous-gene expressions are shown.

CPCs were isolated from the right atria of patients with congenital heart diseases undergoing cardiac surgery (Figure 1A). Individual cardiosphere-derived CPCs were isolated and infected with a combination of retroviruses encoding the transcription factors OCT4, KLF4, SOX2, and MYC as previously reported (Figure 1B) [11]. Among the iPS clones generated, five HLHS-iPS clones (iPS30, iPS46, iPS59, iPS68, and iPS72) and one BV-iPS clone (iPS65), obtained from a total anomalous pulmonary venous connection (TAPVC) patient, could propagate robustly when maintained on SNL feeder cells. We then used the iPS clones (iPS30-, iPS46-, and iPS72-HLHS) and the iPS65 clone (iPS-BV) for further evaluation in this study. Genomic integration of viral transgene was confirmed by RT-PCR. The reactivation of endogenous genes *OCT4* and *NANOG* led to higher levels in patient-specific iPS cells than in parental CPCs (Figure 1C). We also examined the expression of viral transgenes; RT-PCR showed that four factors (transgenes) were efficiently silenced in the established iPS cell lines; they were maintained for more than 15 passages and examined at 3 months post-infection (Figure 1D).

### Characterization of Patient-Derived iPS Cells

**Figure 2:**
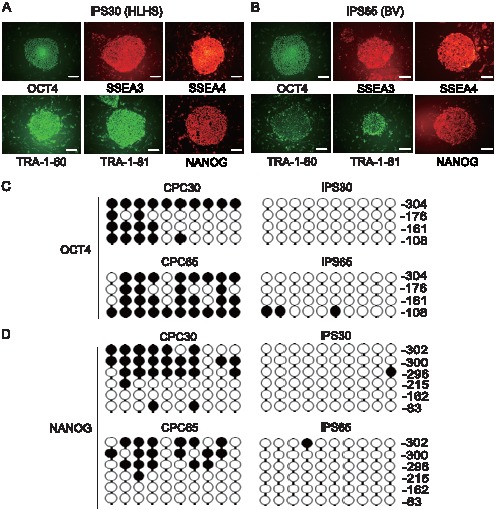
Characterization of disease-specific iPS cells. Representative patient-specific iPS clones at passage 10 (A, iPS30: HLHS; B, iPS65: TAPVC, representing BV). Colonies were stained with transcription factors typically expressed in iPS cells. Bar, 200 µm. (C and D) Bisulfite sequencing analysis of *OCT4* and *NANOG* promoter regions during reprogramming is shown. Closed and open circles represent methylated and unmethylated CpG dinucleotides, respectively.

**Figure 3:**
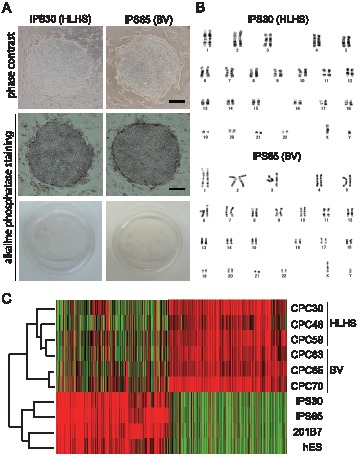
Patient-specific CPCs were fully reprogrammed. (A) Representative images of alkaline phosphatase staining are shown for iPS cells generated from HLHS (iPS30) and TAPVC representing BV (iPS65) patients. Bar, 200 µm. (B) Chromosomal abnormalities were not found in both iPS clones at 10 weeks by the G-banding method. (C) Heat map (right) and hierarchical cluster analysis (left) of global gene expression from patient-specific CPCs and iPS clones are shown. A commercially available 201B7 clone (Riken) was used as control human iPS cells.

The two types of disease-specific iPS cell grew at similar rates and uniformly expressed stringent pluripotent markers such as OCT4, SSEA-3, SSEA-4, TRA-1-60, TRA-1-81, and NANOG, as determined by immunofluorescence (Figures 2A and B). We next sought to confirm the epigenetic reprogramming in individual iPS cells. Bisulfite sequencing analysis was performed to verify the degree of DNA methylation of the *OCT4* and *NANOG* promoters (Figures 2C and D). CpG dinucleotides in both promoter regions were highly demethylated in both patient-derived iPS cells relative to parental CPCs. Consistent with their ES-like morphology, both HLHS- and BV-derived iPS cells were positive for alkaline phosphatase staining (Figure 3A) and could maintain a normal karyotype for at least 10 weeks (Figure 3B).

### Molecular Signatures of Patient-Derived CPCs and iPS Cells

We next performed global gene expression analysis on patient-specific iPS cells and parental CPCs using oligonucleotide microarray (Figure 3C). The heat map image showed that the expression profiles of both iPS cells were similar to those in control human iPS cells (clone 201B7) and human embryonic stem cells (clone khESC-1), but different from that in parental CPCs. Consistent with this, hierarchical clustering analysis demonstrated that disease-specific iPS cells closely resembled control human iPS cells, but were distinct from parental CPCs. Of particular note, pluripotency-associated genes represented by NANOG, POU5F1 (OCT4), and TDGF1 were expressed at remarkable levels in iPS cells compared with the levels in parental CPCs analyzed using three-independent CPC lines (Table S1 in File S1). Patient-derived CPCs significantly expressed typical gene transcripts indispensable for progenitor cell proliferation and differentiation, including IL1B, GREM1, LIF, TGFBR2, and IFGBP7 [12]–[15], and also showed a vascular-lineage-committed phenotype by expressing EDN1, LMO2, and VEGFC (Table S1 in File S1).

**Figure 4:**
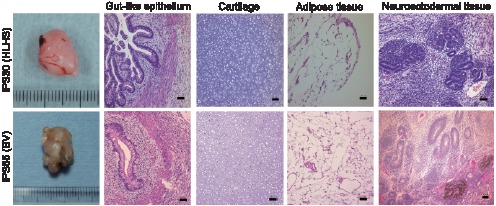
Patient-derived iPS cells differentiated into all three germ layer origins *in vivo*. Gross morphology and hematoxylin and eosin staining of patient-specific iPS cell-derived teratomas are shown. Teratomas were found in the testes of NOD/SCID mice 10 to 12 weeks after transplantation. Histological sections of identified cells represent all three germ layers. Bar, 50 µm.

To assess the *in vivo* pluripotency, iPS cells generated from patients were injected into NOD/SCID mice. Ten to twelve weeks after transplantation, both iPS cells gave rise to teratomas originating from all three embryonic layers, including gut-like epithelia (endoderm), cartilage and adipose tissue (mesoderm), and neuroectodermal tissue (ectoderm) (Figure 4).

### Cardiomyocyte Differentiation Potential of Disease-Specific iPS Cells

**Figure 5:**
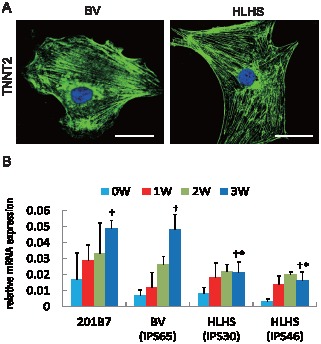
HLHS-derived iPS cells could give rise to cardiomyocytes. (A) Both HLHS- and BV-derived iPS cells could generate cardiac troponin-T (TNNT2)-positive cardiomyocytes (green) 3 weeks after lineage induction. Nuclei were shown by DAPI (blue). Bar, 30 µm. (B) Time course of TNNT2 expression in disease-specific iPS cells. Data were normalized using β2-microglobulin and human heart tissue for comparisons. *, p<0.05 vs. control and differentiated BV-derived iPS cells at 3 weeks. †, p<0.05 vs. before cardiac lineage induction (0 weeks) in each group.

Upon cardiac differentiation, both HLHS- and BV-derived iPS cells generated cells that expressed typical cardiac structural proteins, cardiac troponin-T (TNNT2) verified by immunostaining, suggesting that HLHS-derived iPS cells are capable of generating cardiomyocytes *in vitro* (Figure 5A). Quantitative RT-PCR revealed that TNNT2 expression was significantly upregulated at 3 weeks after differentiation compared with that at baseline in both types of iPS-derived cardiomyocyte; however, HLHS-derived iPS cells showed reduced cardiomyogenic potential than those from control and BV heart (Fig. 5B).

### Reduced Transcriptional Regulatory Programs During Cardiac Differentiation in HLHS-Derived iPS Cells

**Figure 6:**
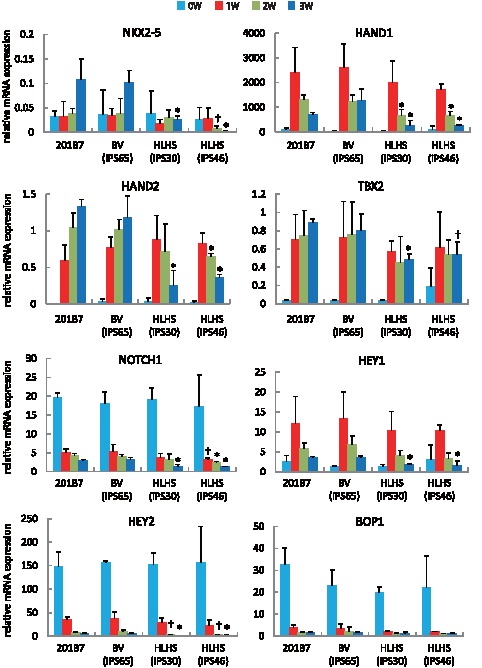
HLHS-iPS cell-derived cardiomyocytes showed decreased cardiac transcripts. mRNA expressions in control 201B7 iPS cells and one BV- and two HLHS-derived iPS cell lines during cardiac lineage induction at respective time points were determined by quantitative RT-PCR. All data were obtained from more than five independent experiments with three different clonal derivatives and normalized using β2-microglobulin and human heart tissue for comparisons. *, p<0.05 vs. differentiated 201B7 and BV-derived iPS cells at corresponding time points. †, p<0.05 vs. 201B7 at corresponding time points.

To determine whether HLHS-derived iPS cells have a distinct cardiac differentiation program, quantitative RT-PCR was performed. We found that cardiac transcriptional factors such as NKX2-5 and HAND1, known to drive cardiac growth and morphogenesis through primary heart field development, were significantly downregulated in HLHS-derived iPS cells at 2 to 3 weeks after differentiation compared with their levels in control 201B7 iPS- and BV-iPS-derived cardiomyocytes (Fig. 6) [16]. *HAND2* gene expression, which is known to preferentially control right heart morphogenesis but has a partially cumulative role with HAND1 in ventricular chamber formation, was also suppressed [17]. T-box transcription factor TBX2, a particularly important regulator for outflow tract cushion development and atrioventricular canal formation as myocardial patterning, was significantly reduced in differentiated HLHS-derived iPS cells [18]. In addition, reduced expression of NOTCH/HEY signaling was found. The decreased transcripts of these genes may be associated with obstruction in the inflow and outflow tracts seen in patients with HLHS due to the developmental defects in the regions of atrioventricular and outflow tract myocardium [19]. These data suggest that HLHS-derived iPS cells have the ability to give rise to cardiomyocytes; however, these cells had suppressed levels of indispensable genes involved in progenitor cell expansion and differentiation to initiate cardiogenesis, atrioventricular canal formation, and left ventricular outflow tract development to achieve functional ventricular growth.

### *NKX2-5*, *HAND1*, and *NOTCH1* Are Indispensable to Restore the Activation of Cardiac-Specific Promoters in HLHS-Derived Cells

Next, we sought to determine whether *NKX2-5*, *HAND1*, and *NOTCH1* genes might be involved in the control of cardiac-specific promoter activities during the development of HLHS. Three possible targets of cardiac-related promoters, namely, SRE, TNNT2, and NPPA, were examined. To demonstrate that the generated shRNAs were specific for *NKX2-5*, *HAND1*, and *NOTCH1*, we performed transient transfection experiments to verify the inhibitory effects of respective gene expressions (Figure S1 in File S1). Four sets of shRNA for each gene were generated and transfected into HLHS-derived CPCs with either the full-length cDNA of interest or combined corresponding shRNA and cultured for 48 hours. Real-time RT-PCR analyses were performed to determine the appropriate ones for subsequent experiments. The inhibitory effects of selected shRNAs were confirmed by using additional clones of HLHS- and BV-derived CPCs (Figure S1 in File S1).

**Figure 7:**
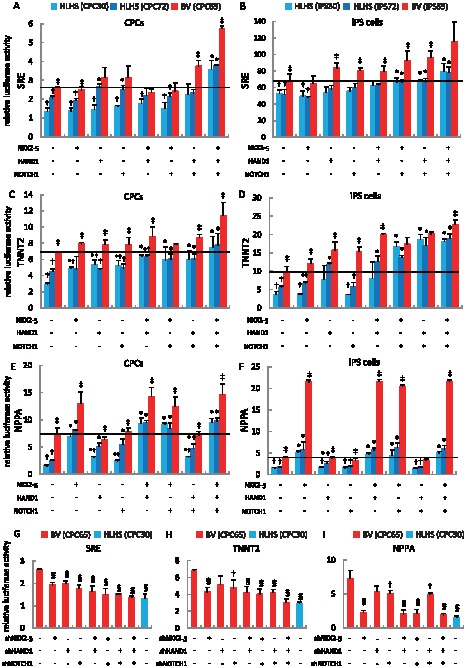
Synergistic restoration of target promoters by *NKX2-5*, *HAND1*, and *NOTCH* in HLHS-derived CPCs and iPS cells. Transcriptional activation of *SRE* promoter luciferase construct by combinatorial transfection of NKX2-5, HAND1, and NOTCH1 in HLHS- and BV-derived CPCs (A) or iPS cells (B). Co-transfection of *TNNT2* luciferase reporter with NKX2-5, HAND1, and NOTCH1 in CPCs (C) or iPS cells (D) is shown. *NPPA* luciferase construct was co-transfected with NKX2-5, HAND1, and NOTCH1 alone or in combination into CPCs (E) or iPS cells (F). (G-I) BV-derived CPCs were transfected with either control or shRNAs specific to inhibit NKX2-5, HAND1, and NOTCH1 expression. Results were normalized using an internal control (SEAP or hRluc) and obtained from more than triplicate sets of experiments. *, p<0.05 vs. the same HLHS sample without transfection of the gene of interest. †, p<0.05 vs. BV sample transfected with control vector alone. ‡, p<0.05 vs. both HLHS samples with the same treatment. §, p<0.01 vs. BV sample transfected with control vector alone.

To investigate whether *NKX2-5*, *HAND1*, and *NOTCH1* might be the crucial transcriptional activators during cardiomyocyte differentiation, we performed co-transfection studies using the luciferase reporters driven by *SRE*, *TNNT2*, and *NPPA* promoters, respectively. As shown in Figures 7A and B, both HLHS-derived CPCs and iPS cells demonstrated a significant decrease in SRE transcriptional activation that was synergistically increased when NKX2-5, HAND1, and NOTCH1 were co-transfected into the cells, which was equivalent to the level in BV-derived cells without exogenous gene induction. To address whether these transcriptional factors are capable of suppressing endogenous SRE activation, we transfected shRNAs into BV-derived CPCs and found that either single shRNA or combinatorial treatment had the potential to suppress SRE promoter activities (Figure 7G). We therefore hypothesized that these transcriptional factors may also participate in cardiomyocyte maturation. A *TNNT2* promoter-driven luciferase reporter was used in patient-derived CPCs and iPS cells with or without exogenous gene transfection. We found that BV-derived CPCs and iPS cells showed prominent *TNNT2* promoter activity compared with those in two independent HLHS-derived cells (Figures 7C and D). Induction of NKX2-5, HAND1, and NOTCH1 synergistically restored the reduced *TNNT2* transcriptional activation in both HLHS-derived CPCs and iPS cells. Loss-of-function studies using shRNAs showed that combinatorial inhibition of three genes, except for shHAND1 alone, could markedly suppress the *TNNT2* promoter activities (Figure 7H). *NPPA* promoter-driven luciferase assays demonstrated a striking finding that either *NKX2-5* alone or combinatorial gene transfection containing *NKX2-5* could fully restore the *NPPA* transcriptional activation in both HLHS-derived cell types (Figures 7E and F). This great impact of NKX2-5 on the *NPPA* promoter was confirmed by shRNA experiments that demonstrated that inhibition of NKX2-5 alone resulted in significant reduction of *NPPA* transcriptional activation in BV-derived CPCs (Figure 7I).

### Histone Modification on *NKX2-5* Promoter in HLHS-Derived iPS cells

**Figure 8:**
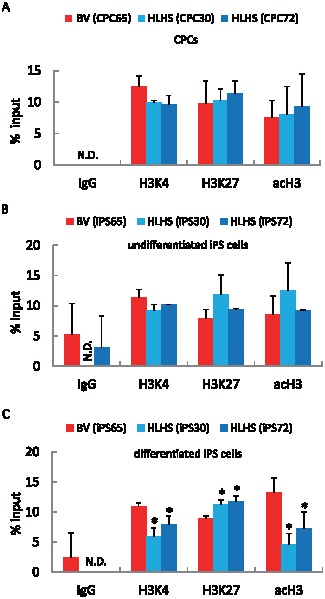
HLHS-iPS cell-derived cardiomyocytes showed suppressed H3K4 methylation and H3 acetylation, but increased H3K27 methylation. Undifferentiated CPCs (A) and iPS cells (B) and differentiated (cardiac-lineage induction for 3 weeks) iPS cells (C) were analyzed by ChIP assay. N.D., not detected; *, p<0.05 vs. differentiated BV-derived iPS cells. Data are expressed as the percentage of input DNA.

To investigate further whether epigenetic modifications, such as histone H3 methylation and acetylation, could be involved in transcriptional regulation in HLHS-derived iPS cells during cardiac-lineage induction, ChIP assay was performed using CPCs, undifferentiated iPS cells, and differentiated iPS cells. Although there were no differences in methylation or acetylation modification at histone H3 in CPCs and undifferentiated iPS cells derived from HLHS and BV patients, a marked decrease in dimethylated histone H3-lysine 4 (H3K4me2) and acetylated histone H3 (acH3) was found within the *NKX2-5* promoter regions in differentiated HLHS-derived iPS cells compared with those from a BV patient. We also identified significantly increased trimethylated H3-lysine 27 (H3K27me3) in the differentiated HLHS-derived iPS cells (Figure 8).

## Discussion

Congenital heart disease involves abnormalities in cardiac structure or function that arise before birth. Although a number of studies have uncovered that heterozygous mutations in cardiac regulatory genes caused congenital heart defects in humans, the identified genetic variants may not be directly correlated with biological insights that potentially contribute to disease development [20]. In lower vertebrates, the key regulatory mechanisms involved in early heart morphogenesis have been investigated extensively, but our understanding of the causal genes responsible for the development of such complex disease is still limited in humans [16]. Recent progress in stem cell biology has revealed a previously unappreciated aspect of cardiac morphogenesis that is genetically controlled by a series of lineage-restriction steps of common progenitor cells that arise from the primary cardiac crescent during development [21]. In this study, we employed an integrated approach by using patient-derived iPS cells to study the pathogenesis of HLHS in order to uncover the molecular fingerprints that may control progenitor cell fate during early cardiac development.

Endogenous CPCs from adult mammalian heart were identified a decade ago [22], [23]. Although human CPCs can also be used in cell culture to dissect the molecular mechanisms underlying congenital heart defects, investigation of inductive signals associated with early cardiogenesis by using postnatal cells, obtained after the onset of the disease of interest, may not be appropriate for the definitive identification of genes responsible for early developmental defects. In addition, it may not be possible to recapitulate the phenotypes by CPCs as *in vitro* sources because the pathogenesis of these complex diseases may require multiple cell types to initiate disease development. Reprogramming technology may facilitate disease investigation by assessing a wide variety of pluripotent stem cell differentiation pathways, including cardiomyogenic commitment, rather than by tracking the lineage-restricted progenitor cell fate. In this regard, patient-specific iPS cells may represent a promising cell source to study disease mechanisms.

There are several limitations in this study. The generation of patient-derived iPS cells remains technically demanding and clonal variation within patients or clones from other patients could be seen among studies; as such, their pathogenetic heterogeneity should not be ruled out. The lack of patients and control samples needs to be further emphasized and acknowledged could be a major limitation in this study. Whether the *in vitro* observations at 3 weeks after iPS cell differentiation could be used as a compatible model of embryonic heart development in humans remain unclear, so the obtained results may need to be interpreted with caution. Ethical concerns have limited the use of human CPCs isolated from healthy individuals due to the safety issues that must be considered during cardiac biopsy procedures. Our approach of using myocardial tissue specimens obtained during cardiac surgery in children was absolutely safe without any appearance of defects compared with the common skin biopsy procedures. With respect to a control, 201B7 iPS cells were used for comparative analysis in this study. We have also isolated disease-derived CPCs from a TAPVC patient in whom the pulmonary veins fail to enter the left atrium but supply the blood flow into the right atrium. Besides the malpositioned pulmonary vessels in this case, four-chamber morphogenesis and outflow tract developed normally in the presence of patent foramen ovale to support oxygenation.

**Figure 9:**
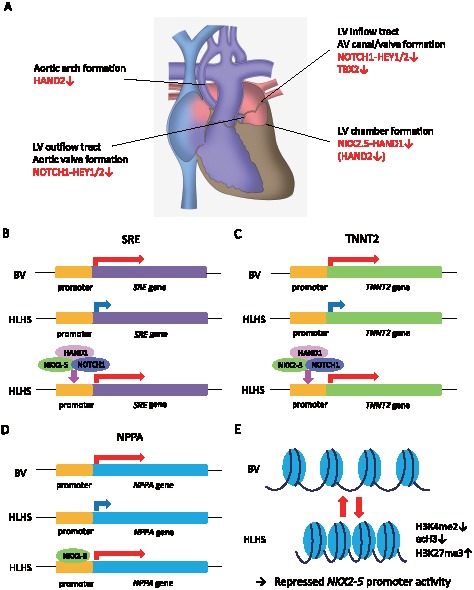
Orchestrated gene regulatory network in the development of HLHS. (A) Core transcriptional factors expressed in cardiac progenitor cells serve as targets in response to inductive signals to initiate cardiogenesis. NKX2-5 is predominantly expressed in the primary heart field and controls progenitor cell proliferation. Genes regulate atrioventricular (AV) canal and valve development. Reduced expression may contribute to mitral- and aortic-valve stenosis/atresia often seen in HLHS. NOCTH modulates left heart outflow tract development and the resultant obstruction may cause secondary ventricle hypoplasia. HAND1/2 specify left and right ventricular chamber morphogenesis, and the absence of these genes may lead to a hypoplastic ventricle. (B–D) Schematic diagrams of *SRE*, *TNNT2*, and *NPPA* transcriptional activation. HLHS-derived CPCs and iPS cells showed significantly reduced luciferase activities compared with BV-derived cells. Co-transfection analysis of reporter constructs with NKX2-5, HAND1, and NOTCH1, proposed core transcriptional factors, could synergistically restore the transcriptional activation in these reporters equivalent to the levels in BV-derived cells. (E) Major chromatin features in differentiated HLHS- and BV-derived iPS cells are shown. Upon cardiomyocyte differentiation, HLHS-derived iPS cells failed to enrich the active histone marks such as H3K4me2 and acH3, whereas repressive histone marks such as H3K27me3 increased, resulting in compact chromatin that lost enhancer marks and gained repressor marks on the *NKX2-5* promoter.

In this study, we found a series of transcriptional repression during the directed differentiation of HLHS-derived iPS cells, which are implicated in the development of HLHS (Figure 9A) and mutually controlled by a core-transcriptional regulatory network, including NKX2-5 and HAND [16]. These results are consistent with a previous mouse study demonstrating that the Nkx2-5-Hand1 transcriptional pathway plays an essential role in left ventricular formation during cardiogenesis [24]. Of particular note, potential *NKX2-5* mutations were found in patients with HLHS [25], [26] and a frameshift mutation in *HAND1* was reported [27]. The epigenetic signature of *NKX2-5* transcripts in iPS cells during differentiation is unknown [28]. In general, undifferentiated stem cells show hypomethylation of specific gene promoters to allow their rapid activation during the processes of differentiation. Methylation at H3K4 is associated with transcriptional activation, whereas H3K27me3 represents a suppressive mark of condensed chromatin status. The results of ChIP assay suggest that reduced H3K4me2 and increased H3K27me3 on the *NKX2-5* promoter might be the alternative epigenetic mechanism to interpret the impaired transcriptional expression found in the differentiation processes of HLHS-derived iPS cells.

The atrioventricular canal is located between atrial and ventricular chamber regions and is an essential source to complete endocardial cushion and valve development. Decreased inflow dynamics may lead to mitral stenosis/atresia as seen in HLHS. Although we did not observe significant changes in BOP1, which is a signaling control of secondary heart field development, combinatorial contributions by the NOTCH/HEY and TBX2 axes from both heart fields might specify this process in HLHS (Figure 9A) [18], [29], [30].

HLHS generally involves a predisposition to obstructed left ventricular outflow, which is commonly associated with aortic atresia [31]. The pathogenesis of HLHS may originate as a primary defect in valve development that leads to secondary LV hypoplasia (Figure 9A). *NOTCH1* mutations have been identified in HLHS individuals and aortic valve anomalies [32], [33]. Recent study related to *NOTCH1* mutations in humans suggests a direct function of activated NOTCH and NOTCH ligand JAGGED complex in controlling myocardial growth through *NKX2-5* activation [34], [35]. The most prominent NOTCH effectors are basic helix-loop-helix transcription factors, HEY1/2. Endocardial NOTCH/HEY signal integration during endocardial to mesenchymal transition has been shown to be critical in the generation of cardiac valve as a specialized structure [36]. Finally, HAND1 and HAND2 are preferentially expressed in primary and secondary heart fields to develop left and right chamber morphogenesis [37], [38]. Although the role of Hand2 is generally essential in the secondary heart field [39], it was reported that the decreased expression of Hand2 in mice could influence formation of the LV and the aortic arch system [24], suggesting some implications in the phenotype of HLHS.

In this study, HLHS-derived iPS cells demonstrated lower capability of differentiating into cardiomyocytes (Figure 5B), which is consistent with a recent report showing that iPS cells generated from HLHS patients had impaired sarcomeric organization as well as altered calcium transient patterning and responses to β-adrenergic antagonist during differentiation when compared with control iPS and human ES cells [40]. These observations indicate that HLHS-derived cells may have critical defects of transcriptional activation that are required for cardiac differentiation and organ morphogenesis of the heart. Among the genes analyzed in this study, *NKX2-5*, *HAND1*, and *NOTCH1* were identified to be the essential transcripts to activate a subset of cardiac lineage-specific gene transcription. In contrast, the transcripts of GATA4 and TBX5 have been shown to be cooperatively involved in directing early cardiac transcriptional activation in vitro and in vivo; indeed, we only observed comparable expression of these genes between HLHS- and BV-derived cells during cardiac differentiation (data not shown) [41], [42]. Novel insights have come from the gain- and loss-of-function experiments that demonstrated that these three transcription factors synergistically regulated *SRE*, *TNNT2*, and *NPPA* transcriptional activation (Figure 9B–D). Although the chromatin states of tissue-specific stem cells have been shown to be intermediate between pluripotent and differentiated cells [43], we found that HLHS-iPS-derived cardiomyocytes but not undifferentiated CPCs failed to acquire active histone marks at the *NKX2-5* promoter region to achieve full cardiac-lineage induction compared with BV-derived iPS cells (Figure 9E). These results suggest that epigenetic pre-patterning during development may also contribute to reduced cardiac-lineage specification and impaired heart morphogenesis in HLHS.

In conclusion, patient-derived CPCs can be efficiently reprogrammed into disease-specific iPS cells for modeling congenital heart malformations. This integrated technology offers an unprecedented opportunity to reveal the genes differentially expressed between iPS cells with and without ventricular chamber defects and might enable correction of the gene variants for therapeutic purposes in HLHS. With knowledge of early cardiac development, the molecular regulatory networks that mediate myocardial growth and morphogenesis can be more informatively dissected by using patient-derived iPS cells.

## Supporting Information

File S1 **File contains Figure S1 and Tables S1 and S2. Figure S1. Verification of inhibitory effects of shRNAs for NKX2-5, HAND1, and NOTCH1 in CPCs.** (A) HLHS-derived CPCs were cultured in growth medium and transfected with transcriptional factors as indicated with or without corresponding four sets of shRNAs. The inhibitory effects for each gene were confirmed by real-time RT-PCR. (B) The most efficient shRNA for each gene was selectively used to inhibit endogenous expression of transcription factors in BV-derived CPCs. Full length of cDNA for each transcription factor was transfected into HLHS-derived CPCs and the repressive effect was examined. Data were obtained from more than five-independent experiments and normalized by using β2-microglobulin and human heart tissue for comparisons. *, p<0.05 vs. sample transfected with gene of interest alone. †, p<0.05 vs. sample without shRNA transfection. **Table S1. Expression of embryonic development-associated genes in patient-derived CPCs and their iPS cell derivatives during reprogramming. Table S2. Primers used for RT-PCR, quantitative RTPCR, bisulfite sequencing analysis, and ChIP assay.**
(DOC)
