# Supplementary material for: Directed Differentiation of Patient-Specific Induced Pluripotent Stem Cells Identifies the Transcriptional Repression and Epigenetic Modification of NKX2-5, HAND1, and NOTCH1 in Hypoplastic Left Heart Syndrome
Source: PLoS One. 2014 Jul 22;9(7):e102796. doi: 10.1371/journal.pone.0102796 (PMC4106834; doi:10.1371/journal.pone.0102796)
Supplement: File S1 — File contains Figure S1 and Tables S1 and S2. Figure S1. Verification of inhibitory effects of shRNAs for NKX2-5, HAND1, and NOTCH1 in CPCs. (A) HLHS-derived CPCs were cultured in growth medium and transfected with transcriptional factors as indicated with or without corresponding four sets of shRNAs. The inhibitory effects for each gene were confirmed by real-time RT-PCR. (B) The most efficient shRNA for each gene was selectively used to inhibit endogenous expression of transcription factors in BV-derived CPCs. Full length of cDNA for each transcription factor was transfected into HLHS-derived CPCs and the repressive effect was examined. Data were obtained from more than five-independent experiments and normalized by using β2-microglobulin and human heart tissue for comparisons. *, p<0.05 vs. sample transfected with gene of interest alone. †, p<0.05 vs. sample without shRNA transfection. Table S1. Expression of embryonic development-associated genes in patient-derived CPCs and their iPS cell derivatives during reprogramming. Table S2. Primers used for RT-PCR, quantitative RTPCR, bisulfite sequencing analysis, and ChIP assay. (DOC) [file pone.0102796.s001.doc]

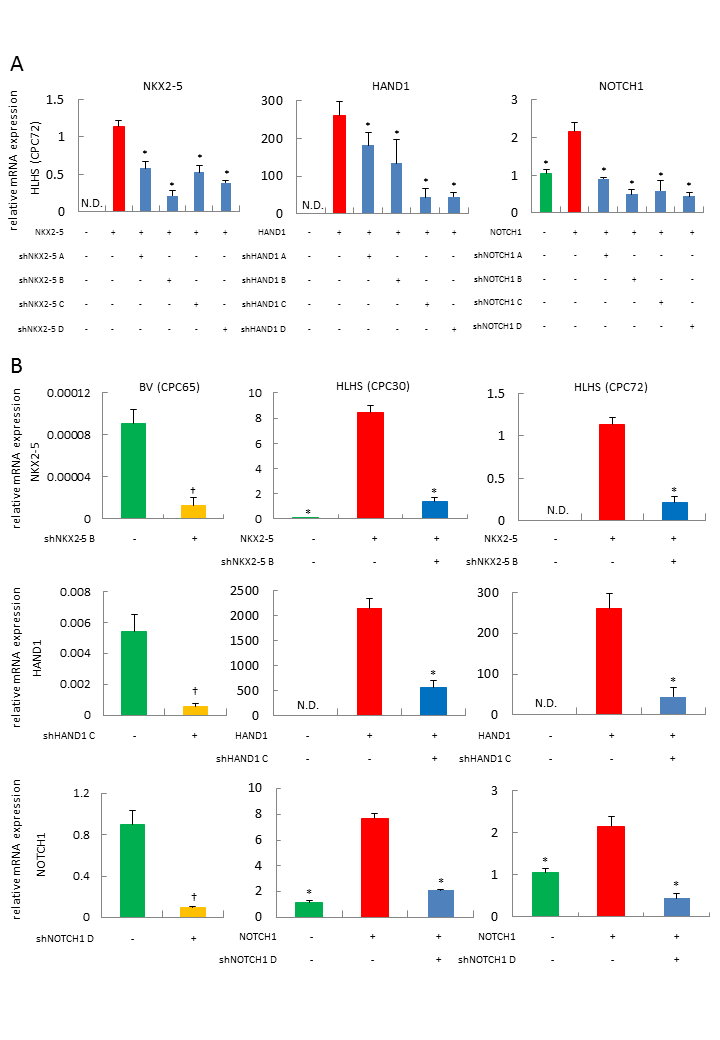
**Figure S1. Verification of inhibitory effects of shRNAs for NKX2-5, HAND1, and NOTCH1 in CPCs.**

**Table S1. Expression of embryonic development-associated genes in patient-derived CPCs and their iPS cell derivatives during reprogramming**

Genes downregulated in patient-derived CPCs compared with the corresponding iPS cells.

| Genbank | Gene symbol | Gene name | Fold change | P value |
| --- | --- | --- | --- | --- |
| NM_024865 | NANOG | Nanog homeobox | 0.01 | 0.0014 |
| NM_002701 | POU5F1 | POU class 5 homeobox 1 | 0.01 | 0.0017 |
| NM_004433 | ELF3 | E74-like factor 3 | 0.04 | 0.0036 |
| NM_002164 | IDO1 | indoleamine 2,3-dioxygenase 1 | 0.06 | 9.77E-5 |
| NM_001878 | CRABP2 | cellular retinoic acid binding protein 2 | 0.06 | 0.0034 |
| NM_020436 | SALL4 | sal-like 4 | 0.06 | 6.78E-4 |
| NM_001040058 | SPP1 | secreted phosphoprotein 1 | 0.08 | 0.0057 |
| NM_003212 | TDGF1 | teratocarcinoma-derived growth factor 1 | 0.08 | 0.0084 |
| NM_000597 | IGFBP2 | insulin-like growth factor binding protein 2 | 0.09 | 0.0093 |
| NM_001109809 | ZFP57 | zinc finger protein 57 homolog | 0.14 | 5.94E-4 |
| NM_031283 | TCF7L1 | transcription factor 7-like 1 | 0.16 | 5.19E-4 |
| NM_001012426 | FOXP4 | forkhead box P4 | 0.18 | 0.0032 |
| NM_002890 | RASA1 | RAS p21 protein activator | 0.19 | 1.91E-4 |

Genes upregulated in patient-derived CPCs compared with the levels in corresponding iPS cells.

| Genbank | Gene symbol | Gene name | Fold change | P value |
| --- | --- | --- | --- | --- |
| NM_006902 | PRRX1 | paired related homeobox 1 | 63.2 | 0.0076 |
| NM_000576 | IL1B | interleukin 1, beta | 60.6 | 0.0039 |
| NM_002581 | PAPPA | pregnancy-associated plasma protein A | 51.4 | 0.0056 |
| NM_000963 | PTGS2 | prostaglandin-endoperoxide synthase 2 | 43.4 | 0.0097 |
| NM_013372 | GREM1 | gremlin 1, cysteine knot superfamily | 38.2 | 0.0021 |
| NM_002309 | LIF | leukemia inhibitory factor | 26.3 | 0.0043 |
| NM_001024847 | TGFBR2 | transforming growth factor, beta receptor II | 24.8 | 0.0075 |
| NM_001553 | IGFBP7 | insulin-like growth factor binding protein 7 | 18.9 | 1.25E-4 |
| NM_002145 | HOXB2 | homeobox B2 | 17.4 | 0.0081 |
| NM_005257 | GATA6 | GATA binding protein 6 | 13.1 | 0.0066 |
| NM_001955 | EDN1 | endothelin 1 | 12.9 | 3.75E-4 |
| NM_005574 | LMO2 | LIM domain only 2 | 12.8 | 9.93E-4 |
| NM_005429 | VEGFC | vascular endothelial growth factor C | 10.6 | 2.02E-4 |
| NM_005529 | HSPG2 | heparan sulfate proteoglycan 2 | 9.3 | 0.0029 |

**Table S2. Primers used for RT-PCR, quantitative RT-PCR, bisulfite sequencing analysis, and ChIP assay**

| Gene | Use | Forward primer | Reverse primer |
| --- | --- | --- | --- |
| OCT4 (endo) | RT-PCR | cctcacttcactgcactgta | caggttttctttccctagct |
| OCT4 (Tg) | RT-PCR | cctcacttcactgcactgta | ttatcgtcgaccactgtgc |
| KLF4 (endo) | RT-PCR | gatgaactgaccaggcacta | gtgggtcatatccactgtct |
| KLF4 (Tg) | RT-PCR | gatgaactgaccaggcacta | ttatcgtcgaccactgtgc |
| SOX2 (endo) | RT-PCR | cccagcagacttcacatgt | cctcccatttccctcgtttt |
| SOX2 (Tg) | RT-PCR | cccagcagacttcacatgt | ttatcgtcgaccactgtgc |
| MYC (endo) | RT-PCR | caacaaccgaaaatgcaccagccccag | gattgaaattctgtgtaactgc |
| MYC (Tg) | RT-PCR | caacaaccgaaaatgcaccagccccag | ttatcgtcgaccactgtgc |
| NANOG | RT-PCR | atgcctcacacggagactgt | agggctgtcctgaataagca |
| GAPDH | RT-PCR | gagtccactggcgtcttcac | ttcacacccatgacgaacat |
| TNNT2 | qRT-PCR | cgaggctcactttgagaaca | ctctgcccgacgtctct |
| NKX2-5 | qRT-PCR | ctaaacctggaacagcagca | cgtaggcctctggcttga |
| HAND1 | qRT-PCR | aactcaagaaggcggatgg | actccagcgcccagactt |
| HAND2 | qRT-PCR | tcaagaagaccgacgtgaaa | gttgctgctcactgtgcttt |
| TBX2 | qRT-PCR | gcacatgctggcatctca | cactagtggcgggcaaag |
| NOTCH1 | qRT-PCR | cgcacaaggtgtcttccag | aggatcagtggcgtcgtg |
| HEY1 | qRT-PCR | catacggcaggagggaaag | gcatctagtccttcaatgatgct |
| HEY2 | qRT-PCR | cccgcccttgtcagtatc | ttgtttgttccactgctggt |
| BOP1 | qRT-PCR | acaacccaccccctgaatac | gcgtggcaaaaagctcag |
| B2M | qRT-PCR | gtgctcgcgctactctctct | tcaatgtcggatggatgaaa |
| OCT4 (1) | bisulfite sequencing | aggaggttggagtagaaggattgtt | aatcccacccactaaccttaacctcta |
| OCT4 (2) | bisulfite sequencing | ggaggggttagaggttaaggttagtg | accccattcctaaccctccaaa |
| NANOG (1) | bisulfite sequencing | gttgtttaggttggagtatagtgg | tataatcccaataaattaaaaaacc |
| NANOG (2) | bisulfite sequencing | ttaatttattgggattataggggtg | aaaaactctatcaccttaaacccac |
| NKX2-5 | ChIP assay | tgtgctcagcgctacctg | ctggcagcttccctgcat |
